# Imaging the Impact of Cortical Microcirculation on Synaptic Structure and Sensory-Evoked Hemodynamic Responses In Vivo

**DOI:** 10.1371/journal.pbio.0050119

**Published:** 2007-04-24

**Authors:** Shengxiang Zhang, Timothy H Murphy

**Affiliations:** 1 Kinsmen Laboratory of Neurological Research, Department of Psychiatry, University of British Columbia, Vancouver, British Columbia, Canada; 2 Brain Research Center, University of British Columbia, Vancouver, British Columbia, Canada; 3 Department of Cellular and Physiological Sciences, University of British Columbia, Vancouver, British Columbia, Canada; Vanderbilt University, United States of America

## Abstract

In vivo two-photon microscopy was used to image in real time dendrites and their spines in a mouse photothrombotic stroke model that reduced somatosensory cortex blood flow in discrete regions of cortical functional maps. This approach allowed us to define relationships between blood flow, cortical structure, and function on scales not previously achieved with macroscopic imaging techniques. Acute ischemic damage to dendrites was triggered within 30 min when blood flow over >0.2 mm^2^ of cortical surface was blocked. Rapid damage was not attributed to a subset of clotted or even leaking vessels (extravasation) alone. Assessment of stroke borders revealed a remarkably sharp transition between intact and damaged synaptic circuitry that occurred over tens of μm and was defined by a transition between flowing and blocked vessels. Although dendritic spines were normally ~13 μm from small flowing vessels, we show that intact dendritic structure can be maintained (in areas without flowing vessels) by blood flow from vessels that are on average 80 μm away. Functional imaging of intrinsic optical signals associated with activity-evoked hemodynamic responses in somatosensory cortex indicated that sensory-induced changes in signal were blocked in areas with damaged dendrites, but were present ~400 μm away from the border of dendritic damage. These results define the range of influence that blood flow can have on local cortical fine structure and function, as well as to demonstrate that peri-infarct tissues can be functional within the first few hours after stroke and well positioned to aid in poststroke recovery.

## Author Summary

The brain is critically dependent on an adequate supply of energy as it consumes up to 20% of the oxygen we breathe. Here we determine the distance scale over which interruptions in blood flow affect synaptic hard wiring and brain function. High-resolution microscopy of live mice was used to image cerebral cortex synapses (the sites of connections between neurons) in real time during targeted interruptions of cortical blood flow that model small survivable strokes. Under normal conditions, synapses were tightly coupled to small brain blood vessels, on average only 13 μm away. During targeted strokes, we find that normal synaptic structure can be maintained by flowing blood vessels at a much greater distance of 80 μm. In contrast to structure, brain function was more sensitive to interruption in blood flow and was only present 400 μm from the border of synaptic structural damage. The identification of intact brain structure in regions lacking function defines brain tissue in which restoration of normal blood flow restores function. Our results define the range of influence that blood flow has on cortical fine structure and function and are important for understanding both the pathology of stroke and how changes in blood flow alter the normal brain.

## Abstract 3

High-resolution structural and functional imaging of the effects of targeted strokes on individual synapses in somatosensory cortex reveals that blood flow from surrounding intact tissue can aid in the immediate post-stroke recovery.

## Introduction

Previous magnetic resonance imaging and histological studies have provided valuable information on the histopathology and the development of ischemic brain damage [1–4]. Based on insight from these macroscopic imaging approaches, therapeutic strategies for stroke involve not saving the ischemic core, but the surrounding penumbral tissues showing partial blood flow or oxygenation [5,6]. Tissues surrounding an ischemic lesion are also proposed to be the site of rewiring and plasticity necessary for recovery from stroke damage [7,8]. Although microcirculation is likely an important determinant of the salvageable stroke penumbra [9], its relationship to synaptic structure and measures of its function remain unclear. To better determine the link between dendritic structure and blood flow, we have imaged individual dendrites and spines of somatosensory cortex neurons in vivo within the ischemic core and surrounding penumbral tissues of transgenic mice expressing yellow or green fluorescent protein. To provide an index of the functional integrity of ischemic cortex, we have mapped somatosensory stimulus-evoked hemodynamic responses using intrinsic optical signal (IOS) imaging [10,11].

Relatively small clot-derived ministrokes, which may be more relevant clinically than widespread damage associated with middle cerebral artery occlusion (MCAO) [12,13], were modeled using rose bengal (RB) photothrombosis [14–16]. RB photothrombosis can produce relatively stable vascular clots [14,16,17] leading to loss of dendritic structure [15]. This model is particularly advantageous to investigate the relationship between cerebral microcirculation and cortical structure since the location, size, and severity of the stroke is controllable. Using the photothrombosis to produce strokes with relatively sharp ischemic borders, we define the scale over which synaptic circuitry and function are supported by local microcirculation. We demonstrate that dendritic structure can be stably maintained by blood flow from small vessels that are on average 80 μm away. Functional assessment using IOS imaging revealed that cortical hemodynamic responses triggered by sensory stimuli (delivered to contralateral limbs) may be present ~400 μm from the edge of structural damage.

## Results

### Assessing the Relationship between Brain Structure, Function, and Microcirculation Using Targeted Strokes

**Figure 1:**
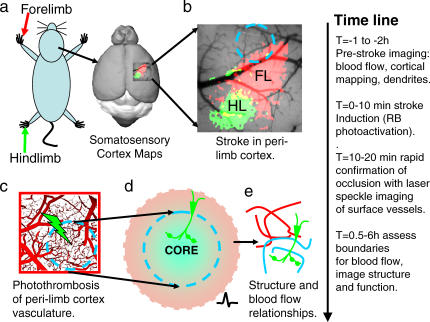
Experimental Setup for Examining the Relationships between Local Vascular Flow and Brain Structure and Function (A) Mice were stimulated by vibrating their fore- or hindpaws while IOS imaging was performed in the contralateral (opposite) cortex to produce somatosensory maps. Maps superimposed on a magnetic resonance imaging reconstruction of a C57 bl6 mouse brain from http://www.bnl.gov/CTN/mouse (at their approximate locations and sizes) [65]. (B) Enlarged view of maps and possible stroke site expected to cause a deficit in the forelimb (FL) map, but to spare hindlimb (HL) function. The point at which map function returns for these ministrokes is termed the functional edge. (C) Boxed region with stylized images of vessels depicting photoactivation of RB by green light. (D) For large strokes up to 1 mm in diameter the ischemic core would contain little or no vascular flow (colored light blue to illustrate ischemia, the dark blue-dashed line is the no-flow boundary). The edges of the core are shown as a mixture of pink and blue to illustrate partial oxygenation due to diffusion from nearby flowing vessels. It is possible that some synaptic structure may be supported within the core by diffusion of blood-derived factors. Outside of the core we will assess where map function is present; note action potential icon. (E) Schematic showing how dendritic structure (green) is assessed relative to flowing (red) and nonflowing (blue) vessels.

Although minimal blood perfusion levels required for brain integrity have been derived using macroscopic imaging techniques [9], events at the interface between normal and ischemic tissues have not been resolved, nor have individual synapses within these regions been followed sequentially in vivo to examine their fate during ischemia. Using two-photon microscopy of yellow or green fluorescent protein-labeled layer V neuron superficial dendrites and 70-kDa Texas Red dextran (TR-dextran)-labeled blood flow, we determined the spatial relationship between cortical microcirculation and synaptic structure and function at the level of individual synapses and capillaries within a targeted RB photothrombotic stroke model in vivo (Figure 1). Strokes were created within 1 mm of the fore- or hindlimb representations within somatosensory cortex to assess their effect on brain function. The limbs were mechanically stimulated to simulate sensory stimuli (Figure 1), and somatosensory response maps were made using IOS imaging.

**Figure 2:**
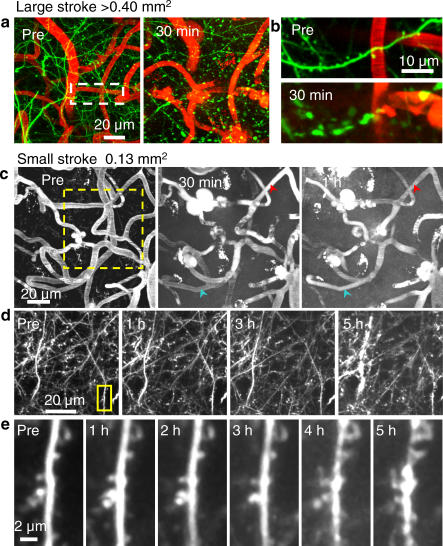
Small Strokes Escape Immediate Damage to Dendrites (A) Images from a YFP mouse showing the vasculature (red) and dendrites (green) (maximal intensity projection of first 100 μm of the cortex) before and 30 min after photoactivation (1 min) of RB in an animal that resulted in severe ischemia and dendritic damage (>0.40 mm^2^ surrounding area with clotted vessels). Vessels labeled with TR-dextran were all clotted in the imaged region. (B) A higher magnification view of the white-boxed region in (A) before and 30 min after photothrombosis. (C) Dorsal view of the microvasculature (maximal intensity projection) from 100 planar scans acquired every 1 μm before, 30 min, and 1 h after photoactivation of RB in a different animal with a small stroke (0.13 mm^2^ of cortical surface with clotted vessels) only affecting a subset of vessels. Flowing vessels can be assessed by the streaked images of vessels reflecting scanning of moving RBCs. The percentage of clotted vessels was 34% and 32% for 30 min and 1 h, respectively. The red arrowhead shows a capillary that was clotted at 30 min and was reperfused at 1 h, and the blue arrowheads mark a capillary that was flowing at 30 min but was clotted at 1 h. (D) Z-projection of dendrites from the yellow-boxed region in (C) showing relatively slow degeneration of dendritic structure for this relatively small stroke with partial reduction in blood flow. (E) Magnified view (yellow box in [D]) showing spine structural changes.

To produce targeted strokes using photothrombosis, RB was delivered by tail vein injection and activated using a green light source over the cortical territories of interest. RB was largely cleared from the vasculature within 10 min (Tau = 175 ± 29 s, *n* = 3 animals) (see Figure S1), reducing the possibility of its ongoing photoactivation by weak two-photon excitation during imaging of TR-dextran and yellow or green fluorescent protein. Consistent with RB photoactivation not occurring in response to two-photon imaging, in animals with RB injection that received no photoactivation (by green light), we did not observe any degradation of dendritic morphology over a 6-h period (see Figure S1) [15]. Using RB photoactivation with green light, clots were formed within 10 min at the center of the photoactivated area in both small and large vessels (Figure 2A and 2C). The area of cortical surface affected by ischemia varied from 200–900 μm in width and extended to 1 mm in depth after 6 h [18]. Previous control experiments indicate that clotted vessels and beaded dendrites were attributed to RB photoactivation since the green activating light alone had no effect [15]. In addition, if RB-induced clots break up and reperfusion is induced, dendritic structural changes can reverse, consistent with the effect being triggered by ischemia and not direct RB photochemistry [15]. Furthermore, RB photoactivation and induction of clotting by a focused laser even within 15 μm of spiny dendrites had no effect if nearby flowing vessels were present (Figure S2).

In 18 of 19 animals, where >90% of vessels were blocked by photothrombosis over >0.20 mm^2^ of cortical surface, widespread blebbing of dendrites and loss of spines occurred within 10–30 min (Figure 2A and 2B). In previous work this damage was found to be reversible in a subset of animals when extensive reperfusion occurred within 20–50 min [15]. In the present study, we have selected animals with relatively stable clots.

As shown here the RB ischemia model can readily produce damage to synaptic circuitry. Advantages of the model are its simplicity and reproducibility, making it amenable to already difficult paradigms such as in vivo two-photon imaging. Although the RB model has certain experimental advantages, a concern is that it may not accurately mirror the histopathology observed with MCAO, a common clinical scenario. Importantly, comparison of MCAO and RB models (Figure S3) indicated that the time course, morphology, and extent of the damage were quite similar, indicating that the changes to dendrites and spines we observed with the RB model are not unique to photothrombotic stroke.

### Slow or Little Dendritic Degeneration in Microstrokes

Contrary to beliefs that photothrombosis models have little penumbral tissue, in 13 animals with smaller strokes (involving <0.3 mm^2^ of cortical surface area), the stroke core contained regions with flowing vessels interspersed with clotted vessels (Figure 2B). Imaging of yellow fluorescent protein (YFP) fluorescence indicated that these partially occluded areas undergo slow or minimal degeneration over a 5-h period (Figure 2C–2E). These and other results (Figure S2) indicate that the presence of clotted vessels alone does not lead to rapidly damaged dendrites provided some blood flow is nearby. Because some animals exhibited vessel occlusion over a smaller area it was possible to estimate at what point the ischemic territories became large enough to affect dendritic morphology. To assess damage within these animals, we used a qualitative five-point rating scale for dendrite damage (see Materials and Methods; Figure S4). After 1 h of relatively stable clotting, we estimated the area of cortical surface with clotted vessels present within the first 100 μm of cortex. We then assessed the correlation between the onset of damage (damage score of 2 or greater) and the area of cortical surface with clotted vessels. This analysis revealed that animals with greater clotted areas reached the onset of damage significantly sooner (*n* = 8 animals; *r* ^2^ = 0.92). Animals having >0.2 mm^2^ clotted areas reached the damage threshold within 30 min, while 0.1 mm^2^ clotted areas took about 3 h to reach threshold. Interestingly, animals with relatively small clotted areas (~0.05 mm^2^ of cortical surface) did not exhibit changes in dendrite structure over 5 h.

### A Sharp Ischemic Border and Localized Ischemic Dendritic Damage

**Figure 3:**
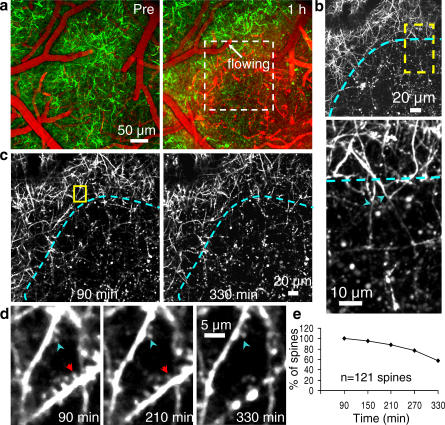
Localized Stroke Damage to Dendrites and Maintenance of Stable and Sharp Borders for Dendritic Damage (A) Low-magnification image of the vascular and dendritic structure before and 1 h after photoactivation of RB. (B) Image showing localized stroke damage to dendrites. Top panel, a magnified view of the white boxed region in (A) showing dendritic structure (a substack Z-projection) 90 min after photoactivation of RB; bottom panel, a magnified view of the yellow-boxed region in top panel showing individual dendrites (arrowheads) that were more damaged and beaded when they projected into an ischemic zone. (C) A magnified view of the yellow-boxed region in (A) showing dendritic structure (a sub-stack Z-projection) at 90 min and 330 min after RB photoactivation. Note the sharp border (blue line) indicates a sharp transition from relatively normal dendritic structure to beaded and swollen structure. (D) Further magnified view (yellow box in [C]) showing a stable spine (blue arrowhead) and a lost spine (red arrowhead) 5.5 h after stroke near the border region. (E) Spine number expressed as a percentage of the number present at the timepoint 90 min after RB photoactivation (it took time to locate the stroke edge, and data prior to 90 min were not obtained for this region).

**Figure 4:**
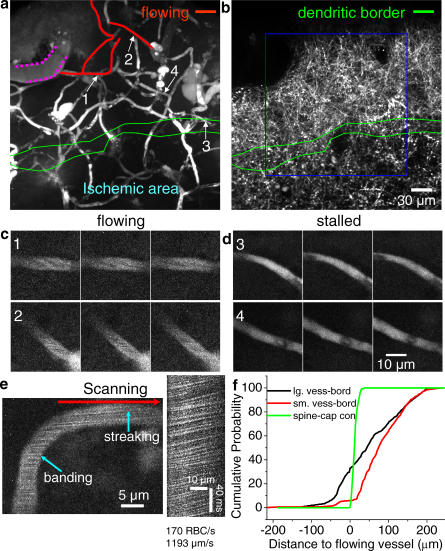
The Relationship between Dendritic Damage Borders and Flowing Vessels (A) Image of the vasculature (maximal intensity projection of first 100 μm of the cortex) 90 min after laser-induced photoactivation of RB. Small flowing vessels are marked in red. The dashed line indicates a large flowing vessel. Green lines denote dendritic damage border as shown in (B). (B) A sub-stack Z-projection (20 μm) showing dendritic structure and dendritic damage border (green lines) 90 min after laser-induced photoactivation of RB. The blue box indicates areas in which dendritic damage borders were examined. Because it was possible that flowing vessels could be located near the edge of an image, a 50-μm buffer zone indicated by the blue line was used to avoid measurements near the border. Distances between the damage border and the nearest small (<15 μm) or large flowing vessel (>15 μm) were made at 10 μm intervals along the green borderline. (C) Representative flowing vessels labeled in (A). Flowing vessels were assessed by determining whether streaking or banding was present (see below). (D) Representative stalled vessels labeled in (A). (E) An example showing vessel streaking or banding that are indicative of objects moving parallel or perpendicular to the direction of scanning (horizontal). The images shown are the average of three consecutive frames, which compresses the width of the bands due to overlap. This single vessel was oriented both parallel and perpendicular to the scan axis and shows that streaks are converted to bands when the angle between the scan direction and the vessel changes (this was also demonstrated by turning the scan angle 90 degrees, not shown). A single linescan through the horizontal part of this flowing vessel is shown on the right showing the velocity and supply rate of RBCs for this vessel. (F) Group data from ten different animals show the cumulative probability distribution of distances between the dendritic damage border and the nearest flowing small or large vessel; to small vessel (*n* = 673 measurements, red line); to large vessel (*n* = 704 measurements, black line) after RB photothrombosis. The cumulative probability distribution of distances between individual spines and the nearest flowing capillary in normal animals (green line) are also shown for comparison; from *n* = 3 animals (250 measurements) from [15]. The cumulative probability distributions were significantly different between groups (Kolmogorov-Smirnov test, *p* < 0.0001).

The ability of small strokes to escape dendritic damage despite the presence of clotted vessels suggested that clotting per se does not have a negative impact providing there was a nearby source of O_2_ and glucose. To better assess the scale over which flowing vessels could support normal dendrite structure, we assessed the relationship between local blood flow and dendritic structure in animals with relatively large strokes where a clear, relatively stable transition between normal and damaged dendrites was observed. To determine the maximal distance over which an ischemic zone can be fed by neighboring perfusing regions (to maintain dendrite integrity), we located the border of the ischemic zone in YFP-expressing mice by monitoring local blood flow. In some animals this distance was difficult to determine since there was not necessarily a sharp transition between flowing and nonflowing regions, or the border region was changing over time. In ten animals that we quantified, we found a remarkably sharp transition between ischemic tissue and perfusing regions within the border region. These animals also exhibited a sharp transition between relatively normal and blebbed dendritic structure that could occur over less than 20 μm (Figure 3A–3C). The effects of stroke damage were highly localized even within a single neuron, since we could observe enhanced damage when individual dendritic processes projected from areas with flowing vessels to ischemic zones (Figure 3B). In this case individual dendrites became beaded when they were well within (~50–100 μm) an area that was largely ischemic (Figure 3B). Within these animals we determined that dendrites of normal morphology could exist in regions with apparently no blood flow, providing an estimate of the range over which local blood flow can support dendritic structure (Figure 4).

To quantify the relationship between damaged dendrites and flowing vessels, we assessed dendritic damage borders at 10-μm intervals within Z-stacks and measured the distance to either the nearest small or large flowing vessel in three dimensions. Whether TR-dextran-filled vessels were flowing was assessed by determining whether streaking or banding was present (see Materials and Methods). Streaked or banded vessel images were indicative of blood cells moving parallel or perpendicular to the direction of scanning respectively (Figure 4C and 4E). If the nearest flowing vessel was found on the intact side of the dendritic damage border, a positive value for distance was given. A positive distance meant that the dendritic damage border was found in an ischemic area. On average, the distance from the border of ischemic dendritic damage (which we term the structural edge determined as in Figures 3B, 3C, 4A, and 4B) to the perfusing edge of blood flow was 84 ± 16 μm (*n* = 10 animals, 673 measurements) for small vessels that were 6.1 ± 1.2 μm in diameter. The borders were measured at 0.75–3 h after stroke when relatively stable clots were present. In some cases the stroke size increased considerably and the experiment was terminated (Figure S5). In five of ten animals at these dendritic structural border regions, we found that large flowing surface vessels were present and defined the border (Figure S5). For large vessels that averaged 29 ± 1.3 μm in diameter the distance to the border of dendritic damage was 40 ± 21 μm (*n* = 10, 704 measurements). It is conceivable that these large vessels in part support nearby dendrites through limited gas exchange [19]. However, since large vessels did not define all borders we do not feel they are an absolute requirement for support of dendrites. It is also likely that the large vessels were either upstream or downstream of smaller flowing vessels. In some cases within the ischemic border region, stable spines were found 30 μm from the structural border region and could be maintained for up to 5.5 h (Figure 3C–3E). Spine counting (*n* = 121 spines) indicated that over a 5–6 h period poststroke, the total number of spines was gradually reduced in regions near the ischemic structural border, suggesting some degree of progressive fine structural damage (Figure 3E). In other cases blood flow deficits became significantly larger (Figure S5), and dendritic damage progressed.

### Little Acute Collateral Damage by Extravasation and Leakage of Serum Derived Factors

**Figure 5:**
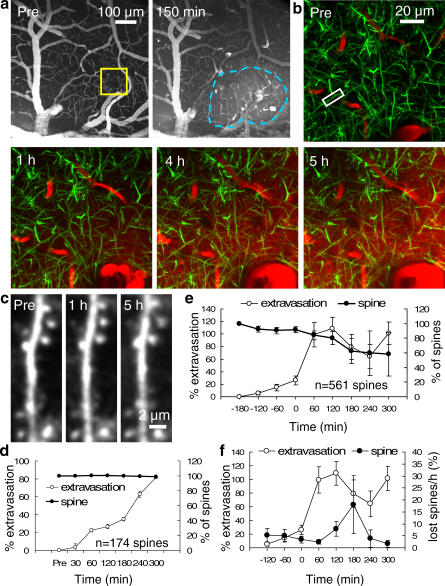
No Relationship between Accelerated Extravasation and Loss of Spines after RB Photothrombosis (A) Low-magnification image of the vasculature (maximal intensity projection, 50 μm) before and after photoactivation of RB. A total of 24% percent of vessels were clotted over an ischemic area of 0.05 mm^2^. (B) Shown are two channel images (a subregion Z-projection) from the boxed region in (A) showing the vasculature (red) and YFP-labeled dendrites (green) before and 1, 4, and 5 h after photoactivation of RB. Vessels were labeled with TR-dextran. The red fluorescence in the tissue indicates extravasation or leakage of plasma containing TR-dextran. (C) A higher-magnification view of a dendritic segment (box in [B]) showing no significant damage or loss of spines despite time-dependent acceleration in extravasation. (D) Quantification of extravasation and spine number over time for this animal. Extravasation was quantified by determining the percentage of vessel TR-dextran fluorescence intensity present in the tissue. Spine number expressed as percentage of the number present before RB photoactivation (*n* = 174 spines). These data indicated that small strokes are not associated with any significant change in spine number over a 5 h period despite a significant increase in extravasation. (E) Group data from nine different animals show relative changes in extravascation and spine number (data aligned by the timepoint when the maximal rate of extravasaton was observed; set as the 0 timepoint) after RB stroke. A one-way analysis of variance indicated a significant effect of time on both spine loss and extravasation. Individual timepoints were compared to the 0 timepoint using Dunnett's post hoc test. We did not observe a significant change in spine number when the rate of extravasation was maximal. (F) There was no significant correlation between the rate of spine loss, change in spine number between consecutive 1-h timepoints, and extravasation at different times (*r* ^2^ = 0.02; *p* = 0.75; nonparametric Spearman correlation coefficient; same data as [E] analyzed differently).

The observation that relatively normal dendrites could be in close proximity to ischemic zones was surprising given the potential for diffusion of potentially damaging substances such as excitotoxic glutamate (released from ischemic zones) to intact tissues. In fact, we observed that 70-kDa TR-dextran used to measure blood flow often leaked from flowing vessels near the ischemic border (*n* = 8 animals quantified) (Figure 5A). To more directly assess the role of potentially diffusible factors from ischemic zones to areas of normal blood flow, we evaluated the correlation between dendritic damage and changes in extravasation (leakage) of blood constituents. Extravasation was scored by determining whether TR-dextran injected into blood vessels leaked into surrounding tissues. Leakage of TR-dextran was quantified as an increase in red fluorescence within tissues (100× tissue fluorescence/fluorescence of a nearby flowing vessel). In some cases, the tissue TR-dextran fluorescence value could exceed that found in large vessels since red blood cells (RBCs) present in vessels strongly absorb two-photon excitation light and thus reduce fluorescence excitation of TR-dextran labeled blood plasma within them.

Surprisingly, enhanced extravasation was not associated with an acceleration of dendritic damage. Figure 5A–5D shows an example where there was no significant dendritic beading or loss of dendritic spines when considerable extravasation occurred, indicating that the two processes can be independent of each other. However, in other animals some time-dependent loss of spines as well as time-dependent increases in extravasation were observed. To further analyze this relationship we increased statistical power by pooling time-dependent extravasation and spine loss data from eight animals by aligning the results to the timepoint when the maximal rate of extravasation occurred for data binned in 1-h timepoints. For these aligned datasets no significant change in spine loss was observed after the point of maximal extravasation by a one-way analysis of variance and post hoc tests (Figure 5E). To further test whether hour-to-hour variations in both quantities were correlated we plotted the rate of spine loss (change in spine number between consecutive 1-h timepoints) versus extravasation at different times and observed no significant correlation between these factors (*r* ^2^ = 0.02, *p* = 0.75; nonparametric Spearman correlation coefficient) (Figure 5F). Assuming the appearance of 70-kDa dextran in tissue reflects the release of plasma constituents, these results suggest that serum-derived factors such as hemoglobin or even glutamate may have little acute effect (over the first 5 h after stroke) on dendritic structure or spine loss during ischemia.

### Functional Changes Associated with Ischemic Stroke

To determine the functional properties both within and at the border of an ischemic region, we assessed the IOS changes attributed to hemodynamic responses associated with hindlimb and forelimb somatosensory function in 17 separate animals. Before stroke, 1 s of 100 Hz vibration delivered to the contralateral hindpaw induced a significant reduction in reflection of 635-nm light that illuminated the somatosensory cortex (Figure S6B top panel, Figure S7D left panel). Maps of stimulus-evoked changes in reflectance were created over 20–70 stimulus presentations and were used to assess hindlimb and forelimb somatosensory cortex function. The initial reduction in reflectance (within the first second) presumably reflects an increase in tissue absorbance due to local production of deoxyhemoglobin associated with increased activity [10,20]. Although the stimuli moved the entire paw and would be expected to elicit sensory and motor activity, the response maps were selective to the contralateral hindlimb territory (distinct from the forelimb map), were reproducible across animals, and were consistent with atlas coordinates for the hindlimb representation [21]. In eight of the 17 animals that showed severe ischemia, the hindlimb and forelimb functional maps were completely abolished within the imaging window (Figure S6B bottom panel) and were rendered undetectable. We monitored spontaneous electrical activity (in four of these animals) by placing a silver wire on the surface of the cortex and embedding it within agarose (Figure S6A). After severe stroke the electrocorticogram (EcoG) was also markedly suppressed in the ischemic core region within animals with severe strokes (Figure S6C).

**Figure 6:**
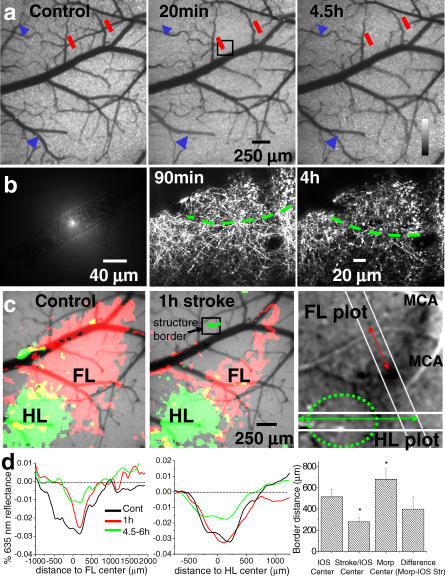
The Relationship between Loss of Dendritic Structure and Activity Dependent Hemodynamic Responses in Animals with Photothrombotic Stroke Targeted to Individual Arteries (A) Shown are laser-speckle surface blood flow images, darker tones indicate lower speckle contrast and higher velocities of blood flow. Left: control speckle contrast image of the right somatosensory cortex (oriented rostral up and medial left). A middle cerebral artery branch and sites where laser induced photothrombosis was performed are indicated by two red lines. Middle: 20 min after targeted photothrombosis blood flow has stopped in photoactivated segment. Blue arrowheads indicate changes in venous blood flow at distant sites. A small black box indicates the location of two-photon imaging shown in (B). Right: 4.5 h after stroke blood flow is still lost within the targeted MCA segment. (B) Image of RB photoactivation location on the MCA branch is indicated by the red line (left-most line). Upstream, to the right of the photoactivation spot a clot can be seen developing. Middle panel: dendritic damage border image taken 90 min after photothrombosis (approximate border indicated by a dashed green line, dendritic damage was reduced below the dashed line). Right: 4 h after photothrombosis dendritic damage worsened, although the approximate border for dendritic damage was in a similar location as that observed at the 90-min timepoint (note the structural transition and some intact dendrites in the lower part of the image). (C) Left: IOS map of contralateral forelimb (FL) and hindlimb (HL) responsive areas (average of 40 trials). Middle: 1 h after stroke the forelimb map has retreated while the hindlimb map is largely preserved (average of 40 trials collected 30–60 min poststroke). Right: raw image of change in reflectance for the 1 h poststroke contralateral forelimb map (images scaled from −0.05% to 0.05% change). The white diagonal and horizontal lines indicate the size and location used for determining response profiles in (D). The locations of MCA branches activated by forelimb stimulation are indicated; the arteries are clearly visible as lighter structures. (D) Quantification of forelimb and hindlimb IOS response from smoothed line profiles indicated in (C) (right). The profiles show a large change in the border of the forelimb territory (left). For the 1-h maps the edge of blebbed dendritic structure was 511 μm from the edge of the forelimb response. The hindlimb functional border was largely unaffected (middle panel). The approximate border locations for IOS responses were maintained for both 1- and 4.5–6-h timepoints. Right: average group data from nine different animals showing distance between the IOS map border and the center of the map for control conditions (column 1) and after stroke (column 2). The distance between the border of morphological dendritic damage and the center of the IOS map is in the third column. The last bar is the difference between bars 2 and 3 for each animal; a measure of the distance between the morphological edge of stroke damage and edge of the IOS response after stroke. Paired t-tests compared column 1 to 2 or 3 (asterisks indicate *p* < 0.02, alpha reduced since two comparisons were performed).

To better assess the relationship between the somatosensory stimulus-evoked functional maps and structural changes to dendrites, we examined a subset of animals (*n* = 9) in which ischemia-induced losses were restricted to only part of the hindlimb or forelimb functional maps (Figure 6A–6D). In these animals we produced local ischemic lesions within 0.4–1 mm^2^ regions of cortical surface that were usually about 1 mm or more from the center of an adjacent hindlimb or forelimb functional map using either a fluorescence arc lamp (*n* = 4) or a laser (*n* = 5), which targeted individual surface arteries. For five of the animals we employed laser-speckle blood flow imaging [22] of large surface vessels to help identify vessels that supply the fore- and hindlimb representations, as well as to confirm the effects of local photothrombosis (Figure 6A). To photoactivate RB (in the example in Figure 6), a 532-nm laser was focused through a 40× 0.8-numerical aperture (NA) objective onto a branch of the middle cerebral artery that ran near the forelimb representation's rostral boundary (Figure 6B). Because of potential for redundant pathways for blood flow (and even flow reversals) [16], we found it necessary to photoactivate RB within at least two points along the arterial branch. Laser-speckle imaging confirmed that the clotting was relatively specific to the targeted arterial segment and could be maintained for at least for 4 h (Figure 6A). Some distant blockade of flow was observed in veins up to several millimeters away, indicating possible diffusion of clots into the venous circulation (Figure 6A, blue arrowheads). In these targeted strokes, we mapped the border of dendritic damage by looking for a transition zone between damaged and relatively intact dendrites at 90 min and 4.5 h after stroke induction (Figure 6B). In this animal the dendritic damage border was in approximately the same place at both timepoints indicating some degree of stability. After targeting the arterial branch, a large component of the forelimb map and rostral and medial forelimb map was missing (Figure 6C).

**Figure 7:**
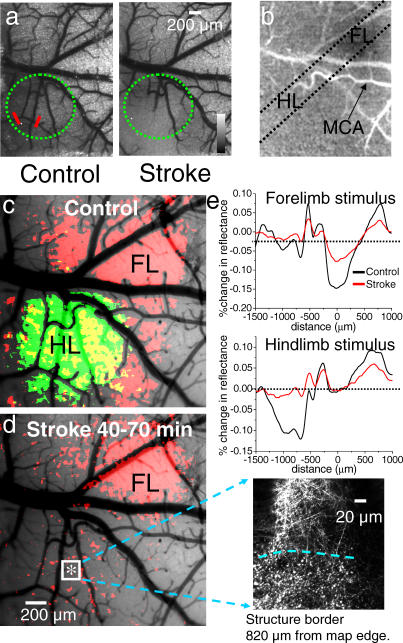
Dendritic Structural Borders after Complete Removal of the Hindlimb Functional Map (A) Laser-speckle surface blood flow image showing the approximate location of the hindlimb territory (green circle) and two sites where photoactivation of RB was performed using a fluorescence arc lamp (red lines). Right: laser-speckle image 20 min after stroke induction shows a loss of surface blood flow to at least half of the hindlimb territory. (B) Raw change in IOS after contralateral forelimb stimulation 40–70 min after stroke indicates a significant forelimb response (darkening in area under FL, images scaled from −0.1% to 0.1% change). In this image branches of the MCA (indicated) that supply both the forelimb and hindlimb area are seen in bright white. This image shows that part of the hindlimb territory is still undergoing changes in blood flow in response to activity. (C) IOS maps for hindlimb and forelimb regions in the animal before stroke induction. The mixed forelimb and hindlimb response presumably reflects some degree of noise (yellow pixels). (D) The hindlimb response was completely lost (no significant response was observed so a color map could not be created) 40–70 min after photothrombotic stroke, while a response in the forelimb area was still present. The white box and enlarged image to the right indicates the area where two-photon imaging established the border of dendritic damage. (E) Quantification of the forelimb and hindlimb IOS responses from smoothed line profiles over the region indicated in (B) by the dotted black lines. Upper panel: contralateral forelimb stimulation results in approximately 0.15% reduction in light reflectance. After stroke reflectance decreases to approximately 0.07%. Lower graph: the contralateral hindlimb stimulus response profile was completely lost following stroke as the red line was not different from 0. The contralateral hindlimb response was visible under control conditions and is observed (in the response profile graph) about −750 μm from the center of the forelimb area.

**Figure 8:**
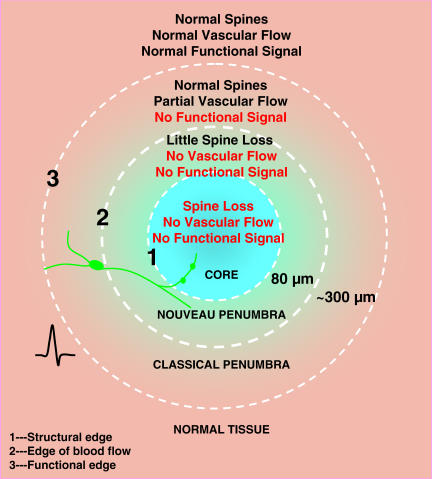
Summary Diagram Showing the Relationship between the Ischemic Core and Synaptic Structure/Function The stroke core in colored blue to indicate poor oxygenation; note the distances shown are not to scale. We find no vascular flow within the core (for small vessels of capillary size), a loss of dendritic structure and spines, and no functional signals (IOS imaging). The first dashed line indicates a region (outside the core) where intact dendrite structure begins to be seen (denoted “1,” termed the structural border). Intact dendritic structure can be found on average ~80 μm from the beginning of blood flow (on the ischemic side of the vascular flow border, vascular flow border denoted “2”). This region, containing intact structure but no vascular flow is termed the Nouveau Penumbra, and represents a newly described region that is ischemic, but has potentially salvageable structure. We have colored the Nouveau Penumbra light blue since it may exhibit partial oxygenation derived from diffusion from areas with vascular flow. Outside of the Nouveau Penumbra is a zone between the beginning of vascular flow and somatosensory function (the functional border is denoted “3”). This region is the Classical Penumbra, which has been previously described, but not resolved at this level. The Classical Penumbra region has partial blood flow and oxygenation, but is nonfunctional. Normal fully perfused tissue lies outside the Classical Penumbra.

As expected we found that contralateral forelimb stimulus-induced changes in IOS were blocked in areas with damaged dendrites (Figure 6B and 6C). What was of more interest was the relationship between the structural transition zone (between damaged and normal dendrites) and the point at which the sensory maps returned. To determine the distance between the approximate edge of ischemic damage to dendrites (structural edge) and the edge of the functional map (the functional edge), we determined the spatial profile of the hemodynamic response. The profile was measured across areas that included the transition between normal and damaged dendrites and continued towards areas with restored function (Figure 6D). We then determined the distance between the edge of the functional map and the map center before and after stroke induction. The functional edge of the map was defined as the point where the IOS response was at 50% of maximal amplitude (defined from a smoothed line profile) (see Figure 6D). As expected, the distance from the map center to the functional edge was reduced on the side of the map that contained a region of ischemia (from 516 ± 73 to 281 ± 38 μm, *n* = 9 animals; *p* < 0.05, paired t-test) (see Figure 6D for an example and group data). In animals where stroke damage reduced sensory maps on the side of the stroke, we asked where the transition between damaged and relatively normal dendritic structure occurred (with respect to the center of the map). In group data after stroke, the edge of dendritic damage was at a significantly greater distance from the map center than the functional edge (edge of dendritic damage 681 ± 106 μm from the map center; *p* < 0.05 paired t-test) (Figure 6D). For clarity, we show examples from three different animals (Figures 6B, 6C, 7D, S7A, and S7B). The region between the edge of dendritic damage and the functional border contained relatively normal structure, but little hemodynamic response following sensory-motor activity (see Figure 8 for a schematic). The average difference between the functional and structural edges of ischemic damage was 400 ± 118 μm (Figure 6D). In the example in Figure 6C and 6D at the 1-h timepoint for IOS mapping, the loss of function was selective for a subset of the forelimb region apparently fed by the blocked artery, while hindlimb function was largely unaffected (Figure 6C and 6D). At 4.5–6 h poststroke induction both the hindlimb and forelimb map amplitudes were reduced, although the approximate functional and structural borders were retained.

In another example photothrombotic stroke removed surface blood flow within ~50% of the hindlimb representation's area. The deficit in blood flow was readily visible with laser-speckle imaging and was largest in the caudal region of the hindlimb map (Figure 7A–7C). In this animal the hemodynamic response was completely lost in the hindlimb region. However, dendritic structure was still intact within a large region of hindlimb that lacked an activity-evoked hemodynamic response (Figure 7D inset). Function, as assessed by IOS mapping, was only present in the forelimb area, with the distance between the dendritic structural border to the functional border being 820 μm (Figure 7B, 7D, 7E).

Our group data indicate that small flowing vessels were on average 84 ± 16 μm (*n* = 10 animals) on the nonischemic side of the dendritic damage border (Figure 4F). Therefore, since the functional edge was on average 400 μm on nonischemic side of the dendritic damage border we would expect a ~300-μm transition zone with flowing vessels and intact dendritic structure, but with little activity-evoked hemodynamic response (Figure 8 for schematic). The presence of this transition zone would suggest that activity-evoked hemodynamic responses would not be limited by the availability of blood flow. Although, the findings above reflect the average results of nine animals (map function ~400 μm from damaged structure), in some animals the hindlimb hemodynamic-response border was less than 200 μm from the area with damaged dendrites (Figure S7). This result indicated that IOS maps can be measured relatively close to areas with dendritic damage. In total, these results indicate that relatively intact functional circuits can exist near regions with extensive damage, and that peri-lesion tissue would be likely to play a role in recovery of function.

## Discussion

### Dendrites Are Maintained at the Border of Ischemic Zones by Distant Blood Flow

Our previous data indicated that in superficial mouse somatosensory cortex, dendritic spines were on average 13 μm from a capillary [15]. These data are supported by independent measurements in nonhuman primates that reported a 16 μm neuronal soma to capillary distance in the normal basal ganglia [23]. By measuring the distance from the edge of blood flow to the structural stroke border, we found spines can be maintained in largely ischemic areas by flowing vessels on average 80 μm away. This result suggested that normal blood flow is more than sufficient to maintain synaptic structure and provides a buffer to reduce perturbation. The distance between flowing vessels and damaged dendrites may represent the limit of oxygen and nutrient support from a distant vessel to a neuron or its processes. These data are consistent with postmortem histological observations showing that neurons at different distances from microvessels are differentially affected by ischemia [23]. Our measured distance (~80 μm) over which structural integrity is maintained by distant flowing vessels was similar to that predicted (30–40 μm) from a more general experimental model for tissue perfusion [24]. Perhaps, the deviation between these values and what we observe may be related to astrocytes or other mechanisms that expand the diffusion limit by providing alternative metabolic substrates such as lactate [25]. In future studies it may be possible to use amplified laser pulses to clot individual deep vessels and create even more defined strokes that specifically model different components of stroke including hemorrhage and extravascation or block individual penetrating arteries that may form a bottleneck for local circulation [17,26].

In ischemic border regions damage was quite localized and the ischemic penumbra can be relatively stable and in some cases not subject to spreading damage from ischemic zones despite apparent loss of 70 kDa TR-dextran, suggesting mechanisms exist to confine damage to these regions. Although microglia that are activated by various injuries and diseases (including ischemia) are the cause of potentially deleterious inflammation, they are also potential candidates to restrict the spread of damage [27,28]. Recent data indicate that microglia provide a protective shield preventing the brain from focal blood-brain barrier disruption [29,30]. Astrocytes are another potential candidate for limiting spread of damage and have been shown to be neuroprotective both in vivo and in vitro [31–34]. Generally, astrocytes are more resistant to ischemia, and they can protect neurons from ischemic damage [25,35,36]. In addition to potential cellular mechanisms for restricting dendritic damage, the sharp borders may also be caused by a metabolic threshold defined by the spatial properties of local O_2_ and glucose diffusion. We do not believe that the sharp borders of structural damage we observe are unique to the RB ischemia model since we also examined markers for cell death at the stroke border in five C3H mice 24 h after a permanent MCAO and observed that flurojade staining (a marker for dying cells) decreased with a half-width of 50 ± 8 μm (unpublished data).

The presence of sharp ischemic borders and good correspondence between clotting and damage is particularly surprising given the potential detrimental effect of diffusible toxic substances including: serum contents, free radicals, glutamate, and inflammatory factors [37–39]. One potential complication of the RB model is extravasation (leakage) of vessel contents into the brain parenchyma, which may trigger a relatively large inflammatory response compared to other stroke models [12]. However, spines were even well preserved in regions with apparent extravasation in the RB model (at least within the first 5 h). Therefore leakage of serum-derived factors resulting from extravasation may not be a major cause of acute ischemic damage to dendrites and their spines. It is conceivable that extravasation may trigger inflammation and a worsening of dendritic condition at later timepoints. The benign nature of extravasation would also argue against excitotoxins as being a major cause of acute ischemic damage (in this model) since leaking plasma contains 30–80 μM glutamate [40,41], but apparently this has little effect on the structure of dendrites in vivo. However, our findings do not necessarily exclude a beneficial effect of experimental therapies targeted at blocking glutamate receptors since such strategies may be protective simply by lowering the energy demand in the ischemic area [9,42,43]. Although we show no relationship between accelerated leakage of 70 kDa dextran and spine loss, it is possible that damage-initiating lower molecular weight substances may have been leaked from vessels earlier. As further evidence that extravasation is not a major factor in acute damage to dendrites, in the MCAO model (where we observed little extravasation), dendrites were still damaged suggesting other factors are associated with the acute stroke damage to synapses.

### Relationship between Ischemic Structural Damage and Function

Although functional imaging such as positron emission tomography and magnetic resonance imaging have provided methods to identify ischemic tissue based on regional blood flow, metabolic rate, and other pathophysiologic parameters [44], these methods are limited in their spatial resolution. In our study, IOS imaging of somatosensory-evoked hemodynamic signals [10,11] was combined with two-photon imaging of fine synaptic structure offering a unique opportunity to image both cortical function and fine synaptic structure within the same animals. Using stroke targeted to the border of the contralateral hindlimb or forelimb functional map we have shown that the edge of the functional map is on average 400 μm from the morphological border defined by damaged dendrites. In animals where we abolished part of the hindlimb or forelimb functional map, the remaining poststroke map (even in areas 500 μm from the ischemic border) was usually weaker than the prestroke map. One potential explanation for this is that some horizontal intracortical circuits are lost within the ischemic penumbral region causing areas that are relatively far from the ischemic core to lose synaptic drive. It is also possible that neuronal function in surrounding territories is impaired by lower O_2_ tension due to partially blocked vasculature or by its proximity to O_2_-hungry ischemic territories. An area for future study would be to use lifetime imaging approaches [45] to gauge the level of tissue oxygenation.

To better define ischemic structural changes to synaptic architecture after ischemia it may be possible in future experiments to combine two-photon imaging of brain structure and blood flow (in live animals) with automated serial high-resolution block-face electron microscopy [46], or other automated means of optical histology [47] to reconstruct the ischemic interface after fixation and tissue processing. Furthermore, it is conceivable that presynaptic terminals and axons may also be studied during ischemia with the development of mouse lines that enable strong labeling of defined projections in adult animals [48,49]. Currently, most adult YFP-H or green fluorescent protein (GFP)-M mice have relatively dense dendritic tufts making it difficult to see fine axonal branches in live animals near the surface of the brain. However, using histology we have examined proximal axons as they leave the soma of layer V neurons. We show that axons are even more susceptible than the basal region of the apical dendrite to ischemic damage [18]. Conceivably, some loss of function in areas with intact dendrites could be due to loss of local axonal structural integrity.

Admittedly, although IOS imaging of sensory-evoked hemodynamic signals has relatively good spatial resolution and is noninvasive, it may have limitations. An obvious one is that it is not possible to assess function in areas without blood flow. We do not feel this is a serious limitation since these areas have no spontaneous electrical activity measured by other techniques such as surface EEG (Figure S6). The second concern is that IOS imaging that employs relatively low NA objectives may be sampling a large column of cortex and not just the surface where dendrite structure was assessed. Although this is a concern, the columnar organization of the brain and our observations that stroke damage is radially oriented in the RB and even MCAO models [18], suggests that surface morphology and lack of function are indicative of deeper layers. Perhaps, future studies could assess function with more direct approaches such as voltage-sensitive dyes and local field potentials. However, these approaches have limitations since they are invasive. Voltage-sensitive dye imaging requires removal of the dura (which is typically left intact in our work), and local field potentials involve insertion of electrodes into the brain that will likely influence the local dendritic morphology. It should be noted that these techniques also lack cellular resolution. Even methods such as single unit recordings would not unequivocally pick up signals from dendrites in layer I where imaging was performed and would be most applicable to deeper layer II neurons where cell bodies are present.

Within the stroke core our histology work [18] indicates that damaged dendrites and clotted vessels are found even at the level of layer V cell bodies, indicating that a lack of IOS signal in the core is likely due to dying neurons. After 6 h of RB ischemia, layer V neurons within the core show antibody staining for activated caspases and cleaved spectrin indicating that they may be undergoing aspects of cell death cascades. Within the stroke penumbra, dendritic damage and the incidence of cell death markers fell in parallel (within ~300 μm at 6 h) [18]. Since we observed activity-evoked hemodynamic responses at an average of 400 μm from the edge of dendritic damage, we would expect that these responses are produced by largely structurally intact neurons that lack markers indicative of cell death cascades [18]. One caveat of our histology work is that it uses trapped TR-dextran to map ischemic zones by viewing stable blood clots after perfusion with fixatives. A limitation is that the presence of clots does not necessarily reflect the extent of ischemia since not all ischemic vessels may retain clots following histological perfusion. Furthermore, it is possible that stable clots may have effects on blood flow over a much larger region of cortex than their immediate location would suggest.

We should emphasize that we have typically recorded sensory-evoked hemodynamic responses within the first 2 h after stroke before expansion of ischemic territories occurs as indicated by our histology work using this model [18]. Where possible, we have interleaved two-photon imaging of dendrite structure with IOS imaging trials to ensure that both were taken during a time period when the ischemic borders were relatively stable. It is conceivable that a number of factors could result in time-dependent expansion of ischemic territories including additional clotting caused by vessel damage as well as ischemic depolarization, which have been suggested to propagate damage in other models [50–52]. However, the damaging effects of propagating depolarization may be much more severe in tissues that are also ischemic, since recent work indicates that neuronal swelling in response to depolarization alone is relatively minor when compared to the effects of ischemia [53].

The ability to assay both fine synaptic structure and function within the same animals permitted us to test previous hypotheses concerning the function of penumbral tissues. The classical ischemic penumbra is a morphologically intact region that is partially perfused or oxygenated making it functionally silent [44,54–56]. The presumed basis of the penumbra is that functional and structural integrity have different thresholds of perfusion [9,55]. Our data indicate that this does occur over a ~400-μm wide band of tissue (Figure 8). We also suggest a revision of this concept and a “Nouveau Penumbra”; since we show that synaptic circuitry can exist up to 80 μm within ischemic zones with no blood flow (Figure 8). Other data from our lab suggest that the concept of the ischemic core (an area with no perfusion or intact structure), may even need to be revisited since imaging of dendrites indicates recovery of structure during reperfusion [15], consistent with dendritic damage being reversible under certain conditions [57,58].

We found that the effects of stroke can be relatively contained, permitting functional and damaged regions to exist in close proximity to each other. This finding would suggest that over the 1–3 h (within the treatment window for stroke) collateral ischemic damage can be minimal. Consistent with this we found that intact synaptic structure can even be found ~80 μm within largely ischemic areas, while synaptic function is present at a greater distance ~400 μm from the edge of dendritic damage (Figure 8 shows the relationships between structural edge, functional edge, and perfusing edge). The ability of ischemic penumbral regions to be partially functional also makes them an important target for therapeutic intervention. In the case of small strokes, tissues near the stroke border would be most similar in function to the areas that are lost, giving them a key ability to take over function of lost circuits aiding poststroke recovery [12].

## Materials and Methods

### Transgenic mice and two-photon imaging.

Adult, urethane anesthetized C57BL/6 YFP-H or GFP-M expressing transgenic mice [59] aged 2–5 mo and between 24–32 g were used for all experiments. Details of in vivo two-photon imaging methods have been described previously [15]. Animal protocols were approved by the University of British Columbia Animal Care Committee and were consistent with Canadian Council for Animal Care guidelines. Briefly, anesthesia was induced with urethane (0.12% w/w) and body temperature was maintained at 37 ± 0.5 °C using a heating pad and feedback regulation from a rectal temperature probe. A craniotomy was performed over the right somatosensory cortex while the anesthetized mouse was held by custom-made ear and tooth bars. The skull was fastened to a stainless-steel plate [60] with cyanoacrylate glue and dental cement, which was then attached to 2.54-cm rods that were mounted on an aluminum plate that could be moved on and off the microscope. To minimize movement artifact (due to breathing and heart rate), the exposed brain was covered with 1%–1.5% agarose (Type 3-A Sigma; A9793, http://www.sigmaaldrich.com) dissolved in a HEPES-buffered (pH 7.3) physiological salt solution (in mM): 135 NaCl, 5.4 KCl, 1 MgCl_2_, 1.8 CaCl_2_, and 5 HEPES, and sealed with a custom cut-glass coverslip. We did not measure blood gases or blood pressure in these animals since these measurements require: significantly longer surgery times for implantation of femoral artery and vein catheters (for extraction of blood and replacement of fluid), the use of agents that could affect clotting such as heparin, and the withdrawal of excessive amounts of blood (mouse blood volume is only ~2 ml). It is conceivable that these procedures themselves may impact the results. It is important to emphasize that most of the measurements we make are relative to an unaffected region of cortex. Furthermore, consistent with the targeted nature of RB strokes, we have not found that brain regions that control cardiovascular function are affected during local stroke (no noticeable change in heart rate or respiration during targeted strokes). Assessment of blood oxygen saturation (on average >90%), heart rate (400–600 beats/min), and breathing rate (100–240 breaths/min) in a separate group of animals indicated that under the conditions we used for imaging, physiological parameters were relatively constant over the course of our experiments. All animals were under urethane anesthesia and breathing air. The physiological parameters mentioned above were measured using a Starr Life Sciences Mouse-Ox pulse oximeter (http://www.starrlifesciences.com). Hydration was maintained by intraperitoneal injection of saline (100–200 μl) with 20 mM glucose at 1–2 h intervals. To image blood flow, blood plasma was labeled through a tail-vein injection of a 0.1 ml bolus of 5% (w/v) TR-dextran (70 kDa) (Invitrogen, http://www.invitrogen.com) in PBS [15,60,61]. In some cases to reduce background due to extravasation, the TR-dextran was injected after RB stroke induction.

Animals were fitted into a custom-made two-photon microscope. We performed two-photon excitation with a Coherent Mira 900 Ti-sapphire laser (http://www.coherent.com) pumped by a 5-W Verdi laser and tuned to 910–920 nm to excite YFP or GFP and TR-dextran. Images were acquired by custom software (IgorPro, Wavemetrics, http://www.wavemetrics.com) and by using Olympus IR-LUMPlanFl water-immersion objectives (40×/0.8 or 60×/0.9 NA). In some cases an Olympus super 20× objective was used for low-power mapping only. For in vivo time-lapse imaging of dendritic structure, multiple Z-sections were taken at the indicated time intervals over 5–6 h. The spacing of successive Z-images was 1 and in some cases 2 μm (2 μm for some instances of low power mapping only). All Z-stacks were acquired by averaging of three consecutive frames over 5.5 s per section. The imaged dendrites were typically within 100 μm of the pial surface and in layer I. Images were acquired with a pixel size of 0.137 μm for high-resolution imaging of spines, typically spanning a 140 × 140 μm area. For lower-power mapping of dendritic blebbing and blood flow a 40× objective and a pixel size of 0.2 or 0.3 μm was used.

### Intrinsic imaging.

For intrinsic optical imaging, a large craniotomy (up to 4 × 4 mm) was made over the somatosensory cortex centered over the region of the fore- and hindlimb map representations. The cortical surface was illuminated by red or green light-emitting diodes (LEDs) mounted around the microscope objective driven by a regulated DC power supply (Circuit-test, http://www.circuittest.com). The green LED light source was used for visualizing the surface of the cortex and pattern of vessels. The red LED light source (center at 635 nm) was used for IOS imaging. For imaging of the functional map, the depth of focus was set to 200 μm below the cortical surface. Imaging acquisition was performed using XCAP-standard version 2.2 imaging software (EPIX, http://www.epixinc.com) with a charge-coupled device camera (Sony XC st70, http://www.sony.com) through a 2.5× Zeiss Plan Neofluor 0.075 NA objective (http://www.zeiss.com). We typically imaged an area of 3.09 × 2.3 mm with a pixel size of 4.1 μm [10,11]. Each data-collection session consists of ten trials taken 20 s apart (for both contralateral fore- and hindlimb, trials interleaved). During each trial, 50 control images were collected over 1.67 s and another 50 images were collected after limb stimulation. We integrated eight-bit images in 16 bits (across repeated trials), and then a ratio image depicting 635-nm reflectance following stimulation (over 1.5 s) divided by the prestimulation baseline period (1.5 s) was created with 32-bit resolution. We typically imaged 20 to 70 trials before and after stroke and where possible interleaved IOS imaging and two-photon imaging to assess time-dependent changes in stroke structural and functional boundaries. In some cases a Dalsa M-60 Pantera 12-bit camera (http://www.dalsa.com) was used for IOS imaging and was mounted on a video microscopy setup that employed a short focus front-to-front dual-video lens system as described (3.8 × 3.8 mm field, 7.5 μm pixels) [10,11]. Vibrotactile stimuli were delivered at 100 Hz for 1 s. The image acquisition, limb stimulation, and LED illumination are synchronized using TTL signals.

### Laser-speckle imaging.

Laser-speckle imaging that is based on blurring of coherent laser light-induced scattering by movement of RBCs was performed as previously described [22,62] using a Dalsa M60 Pantera camera mounted on a video microscope. Typically, 400–800 images were collected at 15–30 Hz using 20–50 ms exposures to periodically assess blood flow. For illumination a 635-nm ~5-mW laser or a 784-nm 32-mW laser (Stocker Yale SNF-XXX-785S-35, http://www.stockeryale.com) with an Edmunds Optics Anamorphic beam expander (T47274, http://www.edmundoptics.com) were held directly on a micromanipulator at an angle of 30 degrees and directed at the brain surface that was enclosed by a coverslip and agarose. Individual images of variance were created in ImageJ using its variance filter (5 × 5 pixel area, 3.75 μm/pixel). Following variance filtering all images were averaged, and a single 32-bit image of the standard deviation was produced by taking the square root of the averaged variance image. The standard deviation image was then divided by the mean of all the raw images to help correct for uneven illumination and to create an image of Speckle contrast (standard deviation/mean).

### EcoG recording.

For EcoG recording, a Teflon-coated silver wire (0.125 mm, WPI, http://www.wpi-interconnect.com) was placed on the surface of the cortex within the agarose. The reference electrode was placed on the nasal bone under the skin. The cortical signal was amplified and filtered (0.1–1,000 Hz) using a differential AC amplifier (Model 1700, A-M Systems) and digitized (1,000 Hz) using a 1322A digidata (Axon Instruments, http://www.axon.com). The data were collected using Clampex 9.2 and analyzed using clampfit 9.2 (Axon Instruments).

### Stroke model.

For the RB photothrombosis model, we injected the dye (0.03 mg/g mouse, diluted to 10 mg/ml in HEPES buffered artificial CSF) into the tail vein using a 28.5-gauge needle after warming the tail with a lamp. Photoactivation was performed by exposing the cortex continuously to green light (from a HBO 100-W arc lamp, 535 ± 25 nm; ~3 mW) for 1–2 min using a 10 × 0.3 NA objective to activate the dye. To obtain strokes of variable size using the arc lamp, we varied the field aperture of the epifluorescence source so that the area of light reaching the exposed cortex varied between 0.16 and 2 mm^2^. In some experiments to target individual surface arteries we used a 0.7–1.4-mW (measured at the objective back aperture) 532-nm argon laser (Beta Electronics, MGM-20, http://www.betapointer.com), which was coupled to the microscope's epifluorescence light path and focused into a spot through a 40 × 0.8 NA water immersion lens. For laser illumination individual arteries supplying the forelimb or hindlimb areas were identified from their response during IOS imaging (increase in 635 nm reflected light signal indicating more oxyhemoglobin) and were targeted at multiply points to reduce redundant paths of surface blood flow [16]. Although smaller epifluorescence apertures and laser targeting to individual arteries were associated with smaller strokes it was difficult to predict the size of RB strokes due to the interconnected nature of the rodent surface vessels [16] and potential for the clots themselves or clot inducing factors to diffuse.

For the MCAO model of stroke, ischemia-reperfusion was induced using a modified version of the intraluminal suture method as described previously in rats and mice [63,64]. Briefly, animals were placed in a supine position with the neck extended to facilitate suture placement. A midline skin incision was made along the ventral side of the neck. The right side common carotid artery (CCA) was exposed and ligated using a 5–0 silk suture (Ethicon, http://www.ethicon.com). Then, the distal external carotid artery (ECA) was ligated with two 5–0 silk sutures, and it was cut to half between the two sutures. After the ECA was flipped over, a small hole was cut in the ECA and a 5–0 poly-L-lysine coated Dermalon monofilament nylon suture (United States Surgical, http://www.ussurgical.com) was introduced into the internal carotid artery (ICA) through the ECA to occlude the right MCA. The endovascular suture was pushed into the ICA until a mild resistance was felt, approximately 10 mm from the ICA/ ECA bifurcation and at a position past the junction of the MCA. Then another suture was tightened around the proximal ECA to preventing leakage of arterial blood from the ECA. At this point the suture around the CCA was loosened and the ventral neck skin was sutured. After 30–60 min of ischemia, the skin was reopened, and the suture was withdrawn to restore blood supply. Although ischemia was reliably induced in six animals and confirmed by two-photon imaging of blood flow, we only observed partial reperfusion in all cases.

### Image analysis.

Image analysis was performed using ImageJ software (http://rsb.info.nih.gov/ij).

To reduce photon and photomultiplier tube noise, a median filter (radius, 1) was applied to all images. In some cases assessments of vessel banding and streaking were made from nonfiltered raw images (Figure 4E). Spine counting and blood flow measurement were done as previously described [15]. To assess dendritic damage after RB photothrombosis, we used a five-point rating scale: 0, no damage; 1, blebbed dendrites <5%; 2, 5%–25% blebbed dendrites; 3, 25%–50% blebbed dendrites; 4, 50%–75% blebbed dendrites; 5, 75%–100% blebbed dendrites (Figure S4). Stroke area was measured from maximal intensity projections of TR-dextran-labeled vasculature stacks obtained using an Olympus water-immersion objective (20×/0.95 NA). To simplify blood flow measurements we estimated the fraction of clotted vessels (we only included relatively small vessels and capillaries <15 μm in diameter). Flowing vessels can be assessed by the streaked image caused by scanning of moving RBCs (see Figures 4C–4E, S5C, and S5D for examples). Since some clotted vessels were not always visible after stroke (due to background leakage of TR-dextran), we subtracted the area of these vessels from the prestroke image, and the percentage of clotted vessels was obtained from area of a thresholded image. To measure the distance from the border of ischemic dendritic damage to the perfusing edge of blood flow we typically made the measurements after 1 h, at which time a significant amount of TR-dextran leakage could occur making it difficult to resolve capillaries given tissue background fluorescence. To prevent errors associated with detection of only small capillaries, we examined the distance to the nearest flowing vessel 15 μm or smaller in diameter. This distance was also confirmed using a 3-D measurement approach (see below). Extravasation was quantified as an increase in the TR-dextran fluorescence intensity present in the tissue and was normalized to the fluorescence intensity of the same flowing vessel at each timepoint to compensate for variation in imaging conditions.

To analyze the spatial relationship between local blood flow and the integrity of dendrites after RB-induced stroke, the following procedure was used. In most cases 100-μm thick stacks of somatosensory cortex were taken using 1-μm Z-steps that were the average of three frames acquired over 5.5 s. Flowing and nonflowing vessels were scored by determining whether streaking or banding was present, indicative of cells moving parallel or perpendicular to the direction of scanning (Figure 4E). Typically at least 5–6 Z-sections were made through even small capillaries (over ~30 s) providing a number of opportunities to assess blood flow by examining single sections. In some cases slow movement of RBCs (<5 μm/s) in stalled vessels was observed; this was <200 times less than typical capillary velocity and such vessels were not considered flowing. Tiled stacks in the region surrounding the transition from damaged to relatively intact tissue were made. Stroke damage varied somewhat at different depths so we made 10-μm maximal intensity projections over the region of cortex studied. By making ten maximal intensity projections (for a 100-μm stack) we could derive information concerning the integrity of dendrites as well as flowing and nonflowing blood vessels at different depths. Since it is possible that regions bordering the stack could have intact flowing vessels (which were not imaged), we did not analyze any dendritic damage boundaries within 50 μm of the stroke border in X-, Y-, and Z-dimensions. We then established borders for dendritic damage by assessing where a relatively sharp transition in dendritic structure occurred. Since the spatial relationship between flowing vessels and damage boundaries could vary at different locations in the stack, we made multiple measurements (at 10-μm intervals) of the distance from the dendrite damage boundary to the nearest flowing capillary/small vessel (<15 μm) or large vessel (>15 μm) in three dimensions. The Z-dimension would be accurate to within 10 μm, since this was the section thickness used for the maximal intensity projections. If a flowing vessel was found on the intact side of the dendritic damage border, a positive value for vessel-dendrite distance was given. Conversely, if a flowing vessel was located on the damaged side of the border, a negative value of distance was given. All distance measurements within a stack for a single animal were averaged.

To quantify the IOS changes we used custom written ImageJ plugins. Average ratio images depicting stimulus-induced changes in 635-nm reflectance were calculated from averages of all baseline or stimulation images taken during each session of typically ten trials. The final functional map was based on average ratio image of 20 to 70 stimulus presentations (two to seven sessions). In some cases IOS data needed to be aligned before averaging because of preparation movement. To produce a color map, we thresholded the average ratio image to graphically illustrate the size of the functional map before and after stroke. The threshold was based on 50% of the maximal response measured from a smoothed line profile (moving average of ±10–12 points) across the limb representation. The edge of the functional map was estimated from the position of the 50% amplitude point. For statistical analyses a t-test or analysis of variance were used, unless otherwise noted. Data were expressed as the mean ± the standard error of the mean .

## Supporting Information

Figure S1 Rapid Clearance of RB after Intravenous Injection RB was injected into the tail vein of a YFP-H mouse and two-photon imaging of both red and green fluorescence was started about 2 min later.
(A) Relatively weak RB fluorescence was observed with two-photon excitation ~2 min after injection. Most of the RB signal was gone within 10 min (Tau = 175 ± 29 s).
(B) Time course of RB fluorescence detected by two-photon microscopy using a red emission channel, single 1-μm section shown. In this graph changes in red fluorescence (minus any preinjection offset) divided by YFP fluorescence (to control for small time dependent changes in imaging conditions) are plotted for 6 h. During the first 10 min after injection (when RB levels were highest) only single scan images (1-μm sections) were taken to reduce the chance on-going photoactivation. After this time the brain was scanned repeatedly (at least eight times) at high resolution by taking 100, 1-μm sections as was typically done for timelapse imaging.
(C) High power image of spiny dendrites (a single 1-μm section is shown) before and 6 h after RB injection shows that without photoactivation of RB by green light, little dendritic damage occurs (similar results were obtained in a total of five animals).
(174 KB PDF)

Figure S2 Laser-Induced Photoactivation Does Not Lead to Direct Dendritic Damage (A) Video image showing surface vasculature illuminated by a green LED.
(B) A higher-magnification view of the boxed region in (A) showing both dendritic and vascular structure (merged GFP and TR-dextran image). A RB-containing surface arteriole was photoactivated at several points (green arrows) near where it penetrated the brain. A nearby fine dendritic branch was within 15 μm of the photoactivation area, but showed little acute damage after over 15 min of alternating photoactivation with a focused laser at the three sites. Clots can be seen developing at the three sites. The preservation of dendritic structure was attributed to only partial blockade of blood flow supplying the area of the branch. For creation of the color merged version (only) nondendritic green channel material was manually masked.
(C) Dendritic structure before and 15 min after local RB laser activation.
(256 KB PDF)

Figure S3 Effect of MCAO on Dendritic Structure (A) Ligation of the right CCA unilaterally did not cause dendritic damage within ~5 h within the right somatosensory cortex (necessary to perform MCAO). Red, blood vessels; green, dendrites.
(B) TR-dextran assessment of blood flow indicated a significant reduction in flux and velocity after unilateral ligation of the CCA. The flux for pretreatment was 108 ± 10 RBCs per second, and the velocity for pretreatment was 1,340 ± 230 μm/s.
(C) In another animal unilateral ligation of the CCA followed by suture occlusion of the MCAO lead to rapid and extensive dendritic damage even with significant residual blood flow. Flowing vessels can be assessed by the streaking caused by scanning of moving RBCs. The yellow arrowhead shows a clotted capillary, and the blue arrowhead shows a flowing capillary at 30 min. All vessels are clotted at 105 min in the region shown.
(D) Average blood flow change from three monitored capillaries in the animal after MCAO. The flux for pretreatment was 123 ± 12 RBCs per second, and the velocity for pretreatment was 1,290 ± 80 μm/s.
(E) Area of clotted vessels (expressed as percentage of prestroke total vessels) increased, and spine number decreased with time after MCAO. Within the MCAO model, the percentage of flowing vessels, the RBC flux, and RBC velocity were reduced.
(79 KB PDF)

Figure S4 Dendritic-Damage Rating Scale To qualitatively assess dendritic damage after RB photothrombosis, we used a five-point rating scale: 0, no damage; 1, blebbed dendrites < 5%; 2, 5%–25% blebbed dendrites; 3, 25%–50% blebbed dendrites; 4, 50%–75% blebbed dendrites; 5, 75%–100% blebbed dendrites. Arrowheads show some examples of blebbed dendrites.
(738 KB PDF)

Figure S5 Large Flowing Vessels at the Border of Dendritic Damage (A) Maximal intensity projection of TR-dextran labeled vessels 100 min after photothrombotic stroke induction using a fluorescence arc lamp. In this example TR-dextran was injected after photothrombosis to reduce the incidence of extravasation. Flowing small vessels were colored red. A single large flowing vessel, likely a vein (dashed line), formed the border between relatively intact dendritic arbors on the left and severely damaged structure on the right.
(B) Dendritic damage borders: a 50-μm maximal intensity projection of dendrites is shown. A total of five different dendrite-damage border regions produced at 10-μm Z-intervals are shown in green. We then made measurements of distance between these borders and the nearest flowing small (<15 μm) or large vessel. The blue boxes in (B) indicate areas in which dendritic damage borders were examined. Because it was possible that flowing vessels could be located near the edge of an image, a 50-μm buffer zone indicated by the blue line was used to avoid measurements near the border.
(C) Example of a banded vessel indicated in (A) showing blood flow. A total of three different 1-μm Z-sections are shown through this vessel, and variation in banding pattern in different sections is indicative of robust blood flow.
(D) Example of a stalled vessel in which very little flow and trapped cells are apparent. No streaking or banding pattern was observed.
(E) Laser-speckle blood flow image showing the distribution of ischemia 15 min after photothrombotic stroke induction (dark areas indicate low contrast and blood flow, images scaled from 0%–10% contrast). The area imaged at high power using two-photon microscopy is indicated and is shown to be at the edge of a large ischemic region. Flowing venous branches are observed coming off the large vein. After the dendritic damage border region was found to deteriorate 205 min later, we performed another round of speckle imaging and found even more extensive clotting and that the blood flow border had moved considerably.
(662 KB PDF)

Figure S6 Loss of Functional Maps and EcoG after Severe RB Stroke (A) Video image showing surface vasculature illuminated by a green LED before and after RB stroke. A Teflon-coated silver wire (arrowhead) was placed on the surface of the cortex within the agarose to record an EcoG.
(B) Severe ischemia (2.2 mm^2^ of cortical surface area affected) completely abolished the contralateral hindlimb movement-evoked functional map (darkened area, expressed as a percentage change in the 635-nm reflected light signal averaged over 40 stimulus trials taken at least 20 s apart).
(C) After severe stroke the EcoG was also markedly suppressed.
(414 KB PDF)

Figure S7 Cortical Hemodynamic Responses within 200 μm of Damaged Dendrites after RB-Induced Photothrombosis (A) Contralateral hindlimb stimulus evoked IOS map before and after RB photothrombosis. The map was thresholded and overlaid on an image of the vasculature image.
(B) A two-photon image shows dendritic structure after RB stroke in the blue-boxed region in (A). Yellow line marks the same position in (A) and the dendritic damage border.
(C) Intensity profile across the functional map before and after RB stroke for the red-boxed region in (A). The red arrow indicates the half maximal intensity for the poststroke plot indicating that some cortical function is retained in the structurally intact tissue several hundred μm from the stroke edge. The blue arrow shows the location of the structural edge of the stroke and is also marked by the yellow line in (A) and (B).
(D) Raw IOS signal change in the hindlimb area before and after stroke scaled between −0.1 and 0.1%.
(308 KB PDF)

## Abbreviations

CCA: common carotid artery
ECA: external carotid artery
EcoG: electrocorticogram
GFP: green fluorescent protein
ICA: internal carotid artery
IOS: intrinsic optical signal
LED: light-emitting diode
MCAO: middle cerebral artery occlusion
NA: numerical aperture
RB: rose bengal
RBC: red blood cell
TR-dextran: Texas Red dextran
YFP: yellow fluorescent protein
