# Supplementary material for: Imaging the Impact of Cortical Microcirculation on Synaptic Structure and Sensory-Evoked Hemodynamic Responses In Vivo
Source: PLoS Biol. 2007 Apr 24;5(5):e119. doi: 10.1371/journal.pbio.0050119 (PMC1854912; doi:10.1371/journal.pbio.0050119)
Supplement: Supplementary file 6 [file pbio.0050119.sg006.pdf]

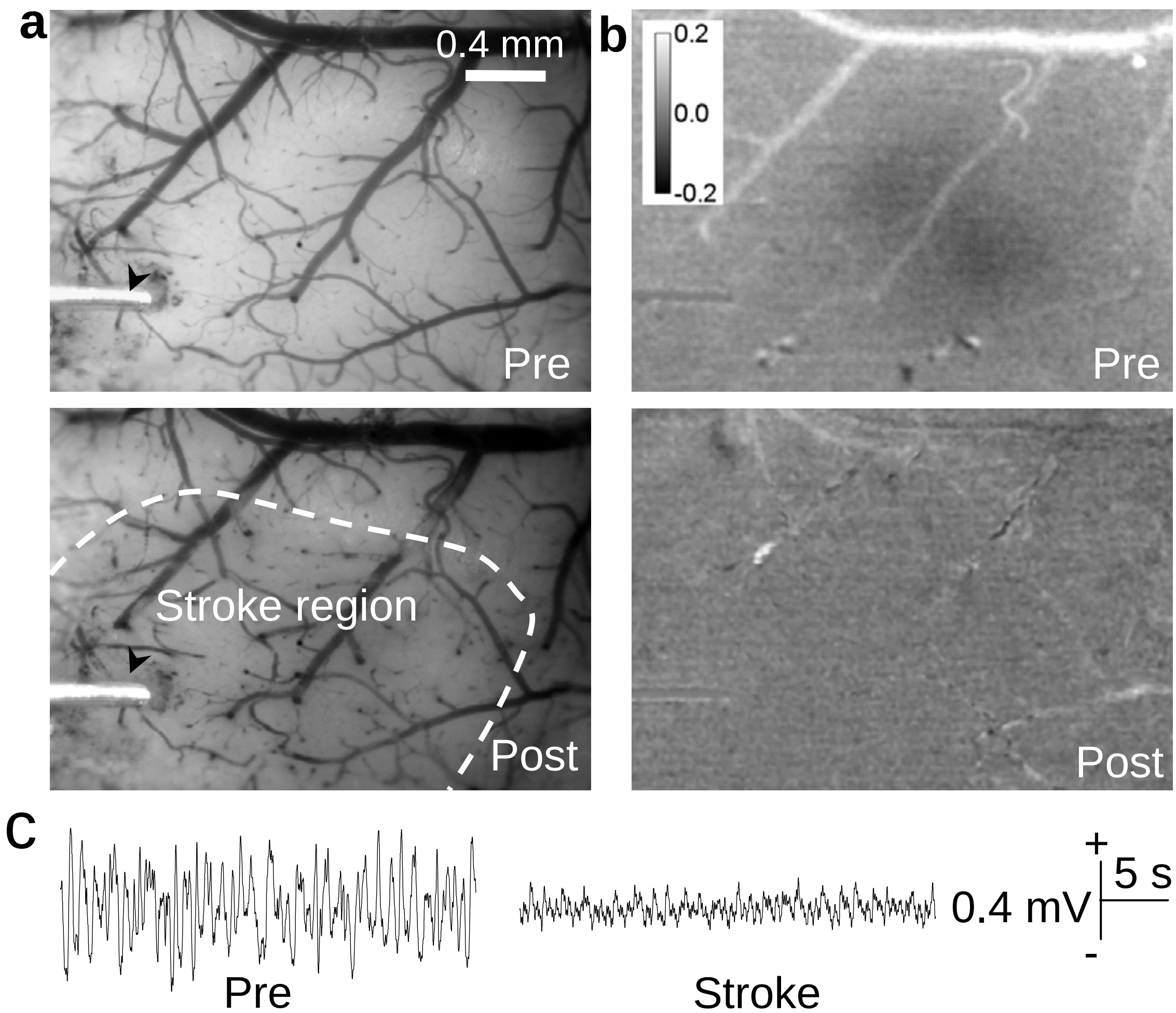

Figure S6_Murphy
